# Immune regulation of the unfolded protein response at the mucosal barrier in viral infection

**DOI:** 10.1002/cti2.1014

**Published:** 2018-04-03

**Authors:** Ran Wang, Md. Moniruzzaman, Eric Shuffle, Rohan Lourie, Sumaira Z Hasnain

**Affiliations:** ^1^ Translational Research Institute Immunopathology Group at Mater Research Institute – The University of Queensland Brisbane QLD Australia; ^2^ Faculty of Medicine The University of Queensland Brisbane QLD Australia; ^3^ Translational Research Institute Inflammatory Bowel Disease Group at Mater Research Institute – The University of Queensland Brisbane QLD Australia

**Keywords:** endoplasmic reticulum stress, epithelial cells, mucosal barrier, unfolded protein response, viral infection

## Abstract

Protein folding in the endoplasmic reticulum (ER) is subject to stringent quality control. When protein secretion demand exceeds the protein folding capacity of the ER, the unfolded protein response (UPR) is triggered as a consequence of ER stress. Due to the secretory function of epithelial cells, UPR plays an important role in maintaining epithelial barrier function at mucosal sites. ER stress and activation of the UPR are natural mechanisms by which mucosal epithelial cells combat viral infections. In this review, we discuss the important role of UPR in regulating mucosal epithelium homeostasis. In addition, we review current insights into how the UPR is involved in viral infection at mucosal barriers and potential therapeutic strategies that restore epithelial cell integrity following acute viral infections via cytokine and cellular stress manipulation.

## Introduction

The endoplasmic reticulum (ER) is a network of branching tubules that extends throughout the cytoplasm of the cell and serves multiple functions. Once protein is translated by ER‐associated ribosomes, it enters into the ER lumen and is folded in a chaperon‐assisted manner. Additional complex modifications occur before the protein is transported to Golgi. Appropriate protein folding and post‐translational modification are crucial for protein function. Aggregated misfolded proteins in the ER cause cellular stress, which if unresolved can lead to cell death. Despite the stringent regulation around protein folding and redundancy within the chaperone‐assisted folding process, both endogenous and exogenous triggers can disrupt the ER homeostasis and increase protein misfolding. These triggers include but are not limited to nutrient deprivation, hypoxia and disruption by chemical inhibitors of polypeptide N‐linked glycosylation (e.g. tunicamycin) or calcium flux (e.g. thapsigargin), oxidative stress and infection. As a result, the ER has evolved a regulatory network, known as the unfolded protein response (UPR), to control the protein folding process. The UPR activation involves three major downstream effects including reduction in protein synthesis to reduce ER load, enhancement of ER protein folding capacity and upregulation of ER‐associated protein degradation (ERAD). If homeostasis is not regained, the UPR will redirect the balance of signalling to favor autophagy or apoptosis. It is increasingly recognised that the evolutionary conserved UPR signalling has an important role in mucosal inflammation and infection. In this review, we discuss the role of the UPR in maintaining mucosal epithelial cell integrity and barrier function and highlight how the UPR is regulated by the host innate immunity.

## The UPR pathways

**Figure 1:**
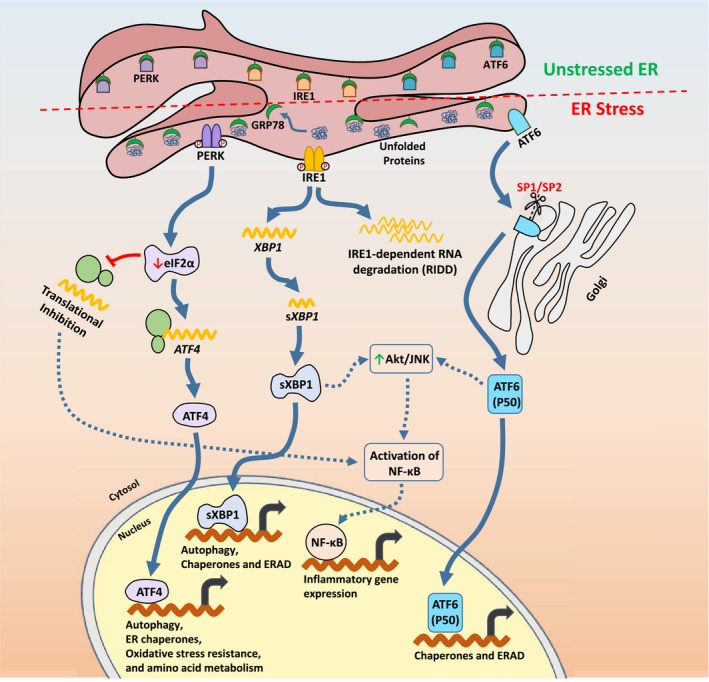
Unfolded protein response. During ER stress, the stress sensors dissociate from GRP78 and transduce signals. Cleaved ATF6 translocates to the nucleus to regulate the expressions of UPR target genes. Activation of IRE1 leads to its phosphorylation and oligomerisation, which induces translation of spliced XBP1 to facilitate protein folding, while long‐term IRE1 activation stimulates RIDD signalling to decrease ER protein folding load. Finally, activation of PERK pathway decreases ER protein load by initiating global translational inhibition through eIF2α. ATF4 gene can escape from the translational suppression and translocate to the nucleus to control expressions of UPR target genes. Moreover, prolonged activation of UPR leads to the expressions of inflammatory genes as shown by the dotted arrows.

The UPR pathways trigger a complex network of signals via three ER transmembrane stress sensors: inositol‐requiring enzyme 1 α/β (IRE1 α/β, also known as ERN1/2), PKR‐like ER kinase (PERK) and activating transcription factor 6 (ATF6), depicted schematically in Figure 1. Under homeostatic condition, the ER luminal domains of these sensor proteins are inactive, due to association with glucose regulating protein 78 (GRP78; also known as BiP). GRP78 has a high affinity for misfolded and unfolded proteins: when luminal load of misfolded protein increases, GRP78 is released from the ER stress sensors, which are then free to initiate downstream signalling outside the ER.

### IRE‐1

The dissociation of GRP78 allows IRE1 dimerisation and activation of C‐terminal endoribonuclease activity, which non‐canonically splices a 26‐base pair intron from the X‐box binding protein 1 (XBP1) mRNA to produce the spliced form of XBP1 (sXBP1). This spliced form of XBP1 then translates into a transcription factor, which further translocates into the nucleus where it induces expression of a wide variety of genes including ER‐associated chaperones and protein folding enzymes to increase ER size and folding capacity. A separate IRE1α‐dependent decay of mRNA (RIDD) pathway is also described (Figure 1).^1^ RIDD degrades mRNAs to ultimately reduce ER load and subsequently reduce the UPR activation. However, with prolonged ER stress IRE1α becomes hyperactive and degrades mRNAs associated with anti‐apoptotic responses, promoting cell death. IRE1β (also known as ERN2) is abundantly expressed by intestine and lung epithelial cells.^2^ Compared to IRE1α, the role of IRE1β in the UPR is not very well studied. IRE1β may also be associated with RIDD,^3^ which is closely related to intracellular parasite infections and anti‐viral responses at mucosal surface.^4^

### PERK

Upon dissociation from GRP78, the transmembrane kinase PERK is activated by oligomerisation and autophosphorylation. PERK phosphorylates eukaryotic translation initiation factor 2α (eIF2α), which inhibits ribosome assembly leading to the inhibition of global protein translation, reducing ER load. Although protein translation is halted under ER stress conditions, translation of specific mRNAs is not subject to inhibition. For example, activating transcription factor 4 (ATF4) escapes translation inhibition, which ultimately leads to expression of genes that regulate amino acid metabolism and autophagy (*discussed later*).^5^ Activated ATF4 in turn induces expression of GADD34, a phosphatase which regulates eIF2α phosphorylation. Once ER stress is resolved, eIF2α is dephosphorylated by GADD34–protein phosphatase 1 complex to restore global protein synthesis.

### ATF6

After sensing misfolding proteins, GRP78 disengages from ATF6α or β isoforms. ATF6 translocates from ER to the Golgi apparatus where it is cleaved by resident proteases sphingosine‐1‐phosphate and sphingosine‐2‐phosphate (S1P and S2P, respectively) to produce a cytosolic ATF6p50 fragment. The ATF6p50 fragment then translocates to the nucleus to modulate gene expression relating to increased ER folding capacity and ERAD pathway activation.^6^, ^7^

The three branches of UPR signalling have reasonably distinct downstream functions, but they are engaged in a coordinated fashion and can act together. For example, XBP1 transcription can be induced by ATF6, and increased IRE1α expression is dependent on PERK‐ATF4 pathway,^8^ suggesting the complex interplay and cross‐regulation between the three branches of UPR pathway (Figure 1).

## Intersection between the UPR and other cellular pathways

Much of our understanding of the role of the UPR in physiology comes from studies where a specific arm of the UPR has been investigated in isolation or the intersection of this pathway with others has not been taken into account. However, there is growing appreciation of the fact that the UPR is interlinked with and may act in concert with other cellular processes such as oxidative stress, inflammation and autophagy.

### UPR‐driven autophagy

Autophagy maintains cellular homeostasis and is involved in MHC class I and II presentation of cytoplasmic and nuclear antigens, which we have reviewed previously.^8^ In response to irreversible ER stress, the terminal UPR can activate autophagy to break down the terminally misfolded proteins. During the autophagy process, cellular components are encapsulated within autophagosomes and directed for controlled degradation. Different UPR branches can signal through and induce autophagy, including PERK‐eIF2α as well as IRE1α pathways (Figure 1). Transcription factor ATF4 that escapes PERK‐associated translation inhibition activates transcription of *CHOP* in cells experiencing persistent ER stress.^5^ ATF4 and CHOP can form a heterodimer to activate cellular death pathways and induce expression of a large array of autophagy‐related genes.^9^

Defects in the UPR and autophagy pathways have synergistic effects. XBP1 is crucial in autophagy induction in some settings, for instance protection from neural degeneration.^10^ XBP1 deletion induces only mild superficial intestinal inflammation; however, concomitant deletion of XBP1 and epithelial‐associated autophagy‐related protein 7 (ATG7) or ATG16L1 results in more severe ER stress and small intestinal inflammation.^11^ Supporting this, human genome wide association studies (GWAS) reveal that polymorphisms in *ATG16L1* gene and defects in autophagy pathways are associated with increased risk of developing Crohn's disease, a form of inflammatory bowel disease leading to chronic inflammation.^12^

Autophagy may also be a mechanism of disposing terminally damaged ER. It was thought to be a non‐specific process, but selective autophagy processes that target specific organelles such as mitochondria have been described. Autophagy of ER in mammalian cells was reported, but detailed mechanisms are still unknown. FAM134B, the selective autophagy receptor for ER turnover, induces selective autophagy of the ER (termed ER‐phagy) in mammalian cells.^13^ It has been suggested that FAM134B binds to microtubule‐associated protein 1A/1B‐light chain 3 (LC3) and GABA type A receptor‐associated protein (GABARAP), thereby initiating ER degradation through autophagy.^13^

### Intersection of the UPR and inflammation

The UPR and inflammation are interconnected on many levels. Defects in protein folding or in any of the individual branches of the UPR spontaneously induce an inflammatory response. In a clinical setting, this has been described particularly in inflammatory bowel disease^8^ and lung disease.^8^, ^14^ Upon sensing pathogen‐associated antigens, pattern recognition receptors (PRRs) including Toll‐like receptors (TLRs) and nucleotide‐binding oligomerisation domain (NOD)‐like receptors can lead to the UPR activation and subsequent inflammation. TLR2 and TLR4 can activate IRE1α with resultant increased sXBP1 required for optimal and sustained production of proinflammatory cytokines in macrophages.^11^ NOD1 and NOD2 signalling can trigger the UPR activation and inflammatory cytokine IL‐6 production from macrophages in a murine model of bacterial infection.^15^ ER stress and the UPR activation generate reactive oxygen and nitrogen species (ROS/RNS), which modify the redox state of the ER, triggering an inflammatory response.

The activation of NFκB, key regulator for immunity and inflammatory response, is linked to the UPR. The NFκB inhibitor IκB has a shorter half‐life than NFκB: activation of PERK and concomitant translational inhibition through eIF2α leads to NFκB activation independent of IκB phosphorylation.^16^ Also, IRE1α can interact with TNF receptor activating factor 2, which recruits IκB kinase leading to IκB phosphorylation and NFκB activation.^17^ It is important to note that ER stress and the UPR have been reported to influence NFκB activation both positively and negatively (Figure 1). The intensity of ER stress influences NFκB activation status positively;^18^ however, preconditioning of cells with low level of ER stress is thought to attenuate NFκB activation.^19^

Conversely, mucosal inflammation modifies ER stress and UPR pathways. Both ROS and RNS produced by innate immune cells during inflammation can activate the UPR in target cells directly. Inflammatory cytokines such as IL‐17A, IFN‐γ and IL‐23 initiate the UPR indirectly via inducing oxidative stress, which inhibits the production of protein disulphide isomerases, that are key components of the endoplasmic reticulum‐assisted folding system (ERAF), resulting in the accumulation of unfolded proteins within the ER and UPR activation.^20^ In contrast, cytokines, like IL‐10 and IL‐22, have been shown to suppress ER stress and its associated UPR activation to alleviate inflammation in intestinal mucosal system.^21^^,^ 21 The evolutionary benefit of the ability of cytokines to rapidly stop/start protein synthesis is unclear, but may relate to cellular defences during viral infection, as discussed later.

## Physiological role of the UPR in maintaining epithelial barrier function

UPR activation plays important role in mucosal homeostasis. Of note, the UPR is involved in cell differentiation and can also be manipulated to accommodate a change in protein load within cells.^21^, ^22^ This is important at the mucosal surfaces where secretory cells must produce large amounts of large, complex proteins to maintain the secreted mucus barrier and provide defence against microbes.

### The UPR activation disrupts intestinal epithelial barrier homeostasis

The intestinal epithelial barrier forms a selectively permeable immunologically tolerant but alert barrier between the sterile inside and microbe‐laden lumen. Mucins, the major macromolecular component of the mucus layer, are complex, highly glycosylated proteins secreted by goblet cells. Due to high secretory demand, the UPR plays an essential role in maintaining secretory cell homeostasis in the intestine. It is hard to delineate the effects of the UPR in isolation, due to the nature of the intrinsically entwined pathways. Consequently, genetically modified experimental models highlight the importance of the UPR in maintaining homeostasis.

Multiple animal models show that the UPR can be pathologically activated at several points when key components such as chaperones, transcription factors or key enzymes are absent or attenuated. Deletion of the major UPR transcription factor XBP1 in intestinal epithelial cells causes loss of mature Paneth cells, reduction in goblet cells, impaired bacterial handling and increased sensitivity to dextran sodium sulphate (DSS)‐induced colitis.^23^ *Ern2* ^*−/−*^ mice lacking ER‐resident endonuclease, IRE1β, have increased Grp78 levels in intestinal epithelial cells and increased Muc2 misfolding,^2^ which lead to increased susceptibility to DSS colitis.^24^ Mice deficient in ATF6 have increased expression of ER stress genes and sensitivity to DSS colitis.^25^ AGR2 is a protein disulphide isomerase involving in disulphide bond formation in proteins within the ER. *Agr2* knockout mice have increased ER stress and suffer from a spontaneous granulomatous ileocolitis with goblet cell depletion.^26^, ^27^

Aside from direct UPR component deficiency, abnormalities in proteins sequence or glycosylation sites also lead to the UPR activation and epithelial cell stress. Single missense mutation in *Muc2* gene leads to misfolding of the major secreted intestinal mucin Muc2, resulting in a strong UPR response and subsequent development of spontaneous colitis characterised by activation of both innate and adaptive immunities with an IL‐23/T_H_17 phenotype.^28^, ^29^ Immune‐regulated alterations in mucin glycosylation following *Trichuris muris* infection contribute to clearance of parasitic infection.^30^

Besides mucin secretion, a new underappreciated role of goblet cells (GCs) is antigen sampling through the goblet cell‐associated antigen passages (GAPs) under homeostatic conditions.^31^ Overriding GC microbial sensing to open colonic GAPs or inappropriate delivery of luminal pathogens through GAPs resulted in the influx of leucocytes and the production of inflammatory cytokines in the setting of normal, non‐pathogenic, microbiota.^31^, ^32^ This microbial sensing by colonic GCs has a critical role in regulating the exposure of the colonic immune system to luminal substances. Although detailed mechanisms are unknown, it presents an intriguing possibility that GC intrinsic UPR and autophagy pathways may be involved in this antigen trafficking process. Interestingly, as a host defence mechanism, GCs are able to shut off GAPs in response to intrinsic sensing of an invasive pathogen like *Salmonella typhimurium* via MyD88 signalling.^33^

Another secretory cell in the small intestine is the highly specialised Paneth cell, which secretes antimicrobial molecules.^34^, ^35^ The UPR plays an important role in Paneth cell production of antimicrobial peptides. Paneth cell‐specific *Xbp1* deletion is sufficient to induce ER stress and autophagy, which results in ileitis which is reversible under germ‐free conditions.^11^ The importance of Paneth cell secreting antimicrobial peptides is highlighted in the bacterial *S. typhimurium* infection model. The UPR activation and associated autophagy are required to ensure antimicrobial peptide secretion from Paneth cells during infection.^36^ Besides the intrinsic UPR activation, extrinsic signals from innate lymphoid cells are also required.^36^ The UPR not only regulates Paneth cell homeostasis, but also preserves its antimicrobial peptide secretory function during infections by increasing the UPR activation threshold to limit pathogen dissemination.

UPR defects within immune cells predispose to inflammation. Lamina propria‐resident dendritic cells (DCs) kill penetrating bacteria by phagocytosis and present bacterial antigens to adaptive T cells. NOD2‐ and ATG16L1‐mediated autophagies are required for DCs to process and present bacteria antigen via major histocompatibility complex (MHC) class II and I to induce CD4^ ^and CD8 T‐cell response.^37^ Individuals carrying *NOD2* and *ATG16L1* polymorphisms display impaired antigen presentation in mucosal DCs and are at increased risk of developing Crohn's disease.^38^, ^39^ XBP1 is constitutively spliced in DCs, highlighting the importance of consistent UPR activation. Loss of *Xbp1* in haematopoietic lineage cells results in a reduced number of DCs. The impairment can be rescued by overexpression of *Xbp1* in haematopoietic progenitors.^40^ Loss of *Xbp1* in CD11c^+^ cells leads to defects in phenotype, ER homeostasis and antigen presentation by CD8α^+^ conventional DCs. These functional defects result from IRE1α‐dependent degradation of mRNAs, which encode MHC class I machinery in the absence *Xbp1,* 41, 42 again highlighting the crosstalk between the UPR and autophagy. Constant IRE1 pathway activation is not exclusive to DCs: developing B cells and T cells also have constitutive activation of IRE1 without activation of any of the other UPR cascades.^43^

### The UPR in airway epithelial barrier homeostasis

Similar to intestinal mucosal epithelial cells, lung epithelial cells have developed many defence mechanisms to deal with environmental exposures. Increasing evidence shows that the UPR pathways interact with the recognition and handling of exogenous threats, like viruses.^44^ The continuous epithelium in the airways acts as a physical barrier to keep the underlying immune system separated from exogenous air‐borne pathogens. Ciliated cells continuously clear inhaled matter trapped by the mucus layer. Respiratory goblet cells synthesise and secrete the mucins, MUC5B and MUC5AC rather than MUC2. MUC5B/AC, and their glycoforms contribute to the elasticity and viscous nature of the mucus layer covering epithelium.^45^ Mice with *Muc5b* deficiency develop spontaneous pulmonary pathology from chronic bacterial infection.^46^ Distinctive to MUC5B, production of MUC5AC is often associated with pathogenesis of respiratory diseases such as asthma or lung fibrosis. In human bronchial epithelial cells (HBECs), sXBP1 induced ER expansion and Ca^2+^ storage contributes to IL‐8 secretion.^47^ IRE1β is expressed in HBECs and is upregulated in cystic fibrosis and asthmatic HBECs. Studies with *Ern2* ^*−/−*^ mice revealed that IRE1β is required for airway mucin production via the activation of the transcription factor XBP1.^48^ In response to ER stress, IRE1β activates its endonuclease activity to repress translation through 28S ribosomal RNA cleavage. IRE1β is abundantly expressed in HBECs;^49^ hence, the IRE1β‐dependent mRNA degradation may be important for efficient translational repression in combating ER stress.

## The immune system dynamically regulates the UPR activation in epithelial cells

Several endogenous factors can activate the UPR and hence alter the protein load of epithelial/secretory cells. However, whether the immune system can have a direct effect on epithelial cells has received little attention. Some studies have demonstrated that inflammatory cytokines can induce oxidative stress within epithelial cells.^50^, ^51^, ^52^ However, studies have mainly focused on epithelial cell‐derived cytokines and/or chemokines rather than cytokines that affect epithelial cells. We demonstrated that specific inflammatory cytokines initiate ER stress by inducing oxidative stress, while other counteracting cytokines suppress stress and facilitate ER protein folding.^53^ While this study focused on pancreatic β‐cells, we have discovered that cytokine regulation of cellular stress is common to multiple epithelial cell types including intestinal and respiratory epithelial cells. In contrast, cytokines, such as IL‐10 and IL‐22, suppress ER stress and UPR activation, leading to increased mucus production, improved barrier function and attenuated intestinal mucosal inflammation in experimental colitis models.^52^, ^54^

**Figure 2:**
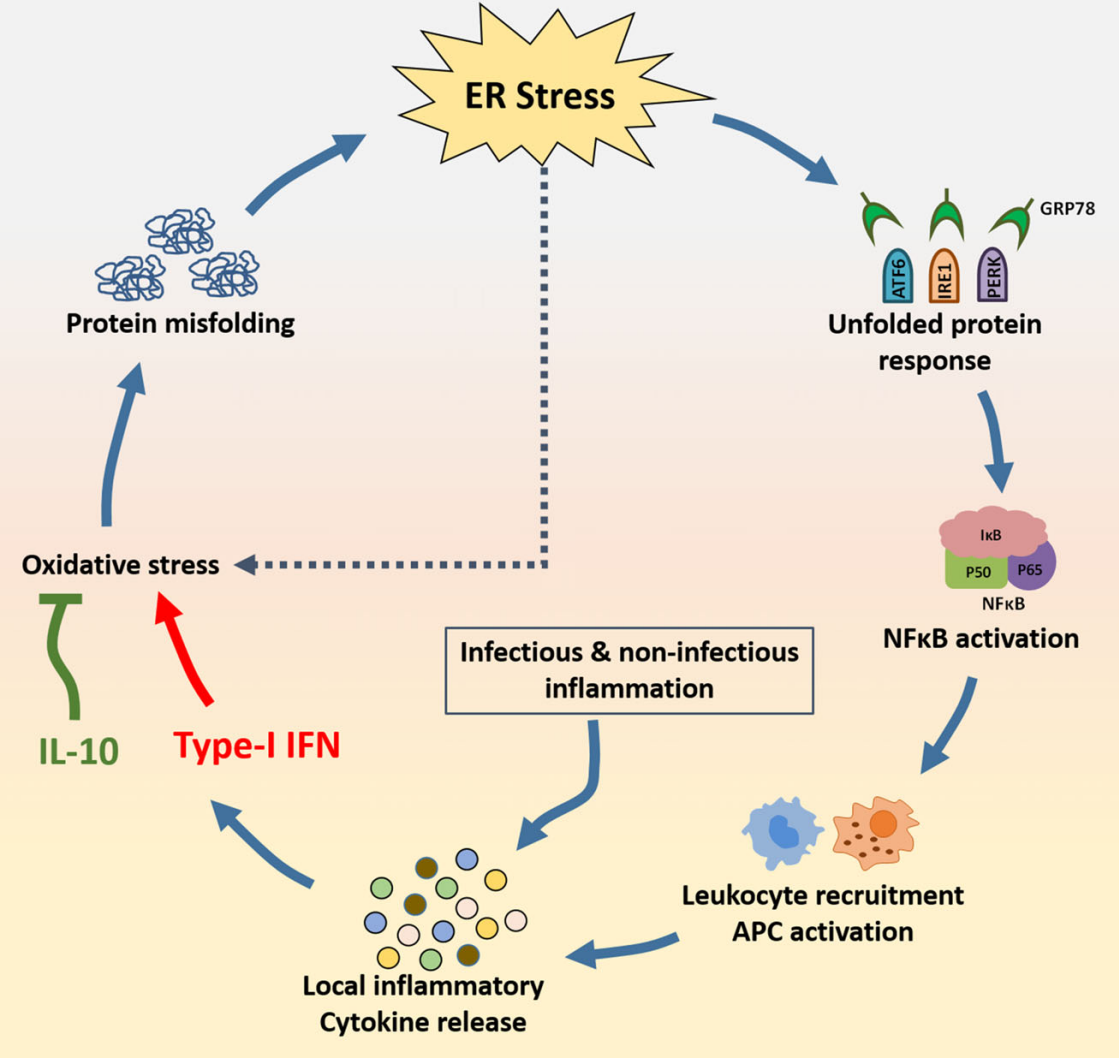
Inappropriate activation of stress cycle. Although the production of cytokines such as interferons (type I) is beneficial for anti‐viral responses; however, prolonged production further induces protein misfolding and leads to a cycle of stress and inflammation in the absence of pathogens. These sequential events destroy the epithelial integrity and leave the epithelium vulnerable to other chronic diseases. Therefore, stress‐reducing cytokines such as IL‐10 can play an important role to minimise protein misfolding and stop stress–inflammation cycle.

**Figure 3:**
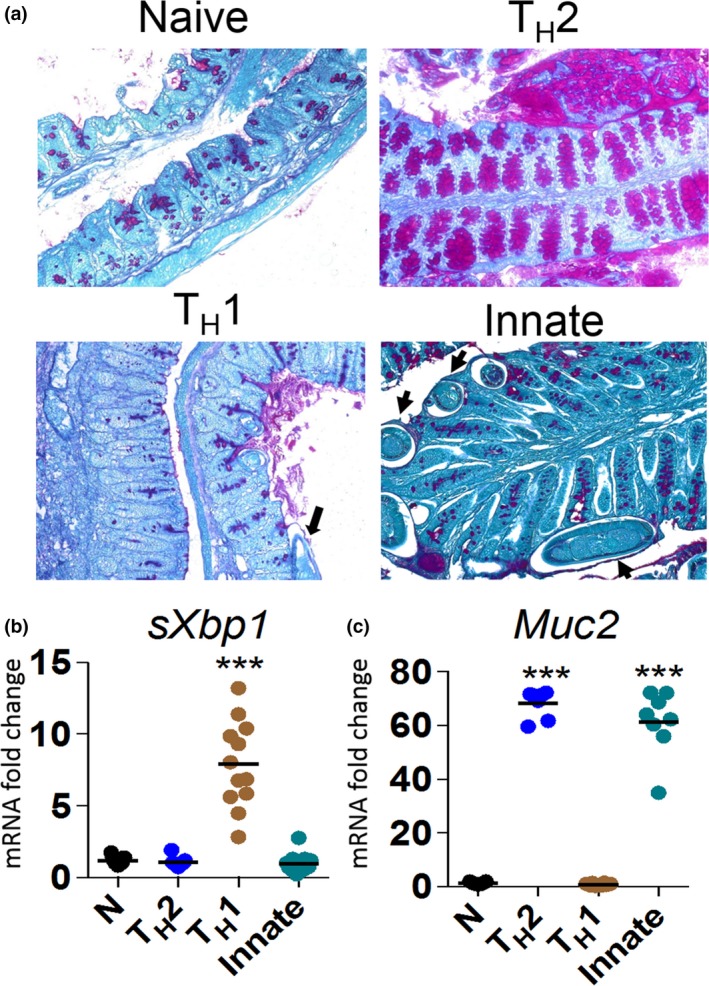
ER stress in mucosal nematode infection. C57BL/6 mice infected with Trichuris muris (T_H_1‐dominant: 15 eggs) or (T_H_2‐dominant and innate: 150 eggs). **(a)** Periodic acid–Schiff staining shows increased protein load within goblet cells, and black arrows indicate the worms. **(b)** Caecal epithelial cell qRT‐PCR shows increased ER stress (sXbp1) in T_H_1 and negligible ER stress in T_H_2‐dominated immune response, despite an increase in **(c)** protein load (Muc2). Immunodeficient mice (innate) show no ER stress despite chronic infection and high protein load. Statistics: *n *= 4–6; ANOVA, ***P < 0.001 vs uninfected (N) mice.

Immunity regulates protein production and secretion by non‐immune cells by indirectly modulating the UPR (Figure 2). As an instructive example of the ability of cytokines to modulate protein production, recent studies in our laboratory show that in chronic infection (nematode, *Trichuris muris*), the amount of ER stress and consequent Muc2 biosynthesis is dictated by an intact adaptive immune response (Figure 3). ER stress occurs in mice that mount a T_H_1/17 response (low‐dose infection), which show goblet cell pathology and fail to clear the infection. However, mice that mount a T_H_2 response (high‐dose infection) show high levels of goblet cell protein biosynthesis and secretion and go on to clear the infection. These data support our hypothesis of specific cytokines modulating protein production. Interestingly, ER stress is not observed in the immunodeficient mice that have chronic *T. muris* infection, strengthening our hypothesis of host intrinsic‐induced cytokines being responsible for the activation of the UPR and induction of ER stress. We suggest that inflammatory cytokines have evolved to disrupt protein biosynthesis in cells prone to viral infection such as mucosal epithelial cells in order to reduce viral replication as a hereto‐unrecognised element of the immune response.

Balanced and integrated ER stress and UPR signalling are crucial in maintaining intestinal mucosal homeostasis, and the immune system can either alleviate or aggravate ER stress.^55^ IL‐10 produced by regulatory T cells can suppress ER stress by inhibiting the translocation of ATF6p50 to the nucleus and downstream p38 mitogen‐activated protein kinase (MAPK) activation in intestinal epithelial cells.^56^ This maintains mucin production in goblet cells, preserving the mucus barrier and reducing further immune activation.^54^ Another regulatory‐related cytokine IL‐22 reduces high‐fat diet‐associated oxidative and ER stress in intestinal epithelial cells and subsequent intestinal inflammation to improve mucosal barrier function.^52^

## The UPR in viral replication at mucosal epithelial barriers

Mucosal epithelial cells, due to their unique location with constant exposure to pathogens, activate different UPR pathways to adapt to the microenvironment and maintain homeostasis. ER stress and UPR activation are mechanisms by which mucosal epithelial cells combat viral infections. Viruses manipulate UPR and host protein synthesis machinery to favor replication. Rather than the chronic UPR activation provoked by inflammation, the UPR pathways are suppressed or bypassed by viruses. Viruses have adapted many ways to hijack the ER to replicate^57^, ^58^, ^59^ and mimic host cytokines/cytokine receptors.^60^ Synthesis of viral proteins often involves high levels of misfolding due to the high mutation rate, the unstable nature of viral genomes and extensive glycosylation of viral envelope proteins.^61^ Moreover, along with viral protein misfolding, induction of ER Ca^2+^ leakage, ER membrane rearrangement and modulation of intracellular glycoprotein trafficking have all been implicated in the UPR response.^62^ Therefore, it is not surprising that viral infections often cause ER stress and activation of the UPR.^59^, ^63^, ^64^

### Viral modulation of the UPR

**Figure 4:**
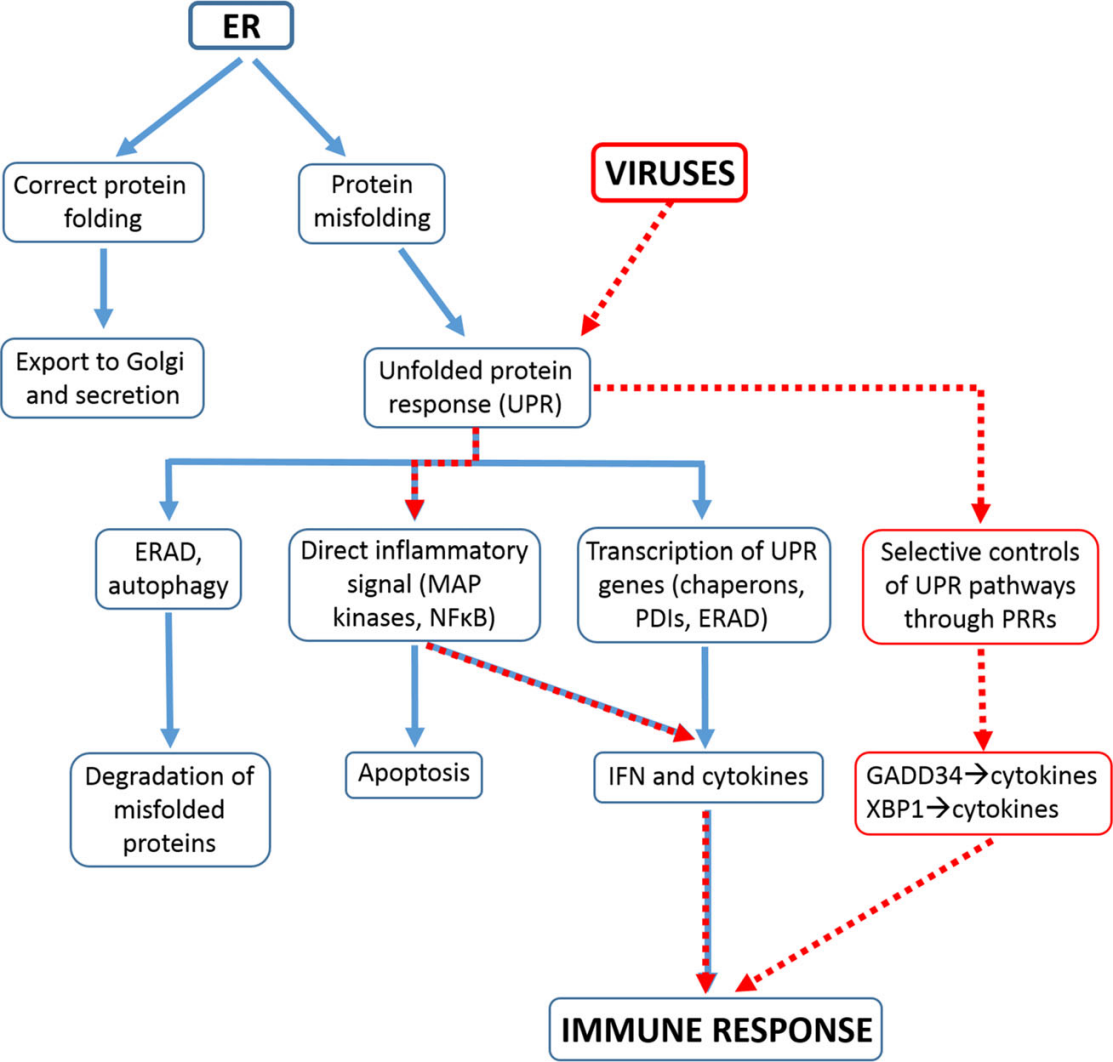
Virus‐controlled UPR response. In homeostatic conditions, proteins are correctly folded in the ER and secreted from cells. However, ER protein misfolding activates UPR pathways leading to the degradation of misfolded proteins, autophagy, inflammation and apoptosis. Viruses can directly or indirectly affect the UPR by selective activation or inhibition of UPR components (shown with red arrows) through endosomal and cytosolic PRRs. Viral‐controlled UPR pathways then ultimately boost the production of viral proteins, while dampening the immune response against the virus.

Viruses have evolved mechanisms to modulate the UPR to either suppress or exacerbate already existing ER stress in order to maximise viral protein biosynthesis, and reduce or increase inflammation (Figure 4, Table 1). Viruses rely on the host ER and the ER stress response to replicate as viral replication is severely diminished in cell lines with mutations in ER‐resident genes.^65^ On the one hand, neither human cytomegalovirus nor West Nile virus, interestingly, induce canonical ER stress pathway via induction of XBP1‐associated gene expression, rather they specifically trigger XBP1 for optimal cytokine production.^66^ Hepatitis C virus, on the other hand, activates IRE1 but inhibits XBP1 and blocks eIF2α phosphorylation to prevent upregulation of ERAD machinery in order to establish persistency in infected hepatocytes.^57^ Some viruses may selectively activate one arm of the UPR while suppressing other branches. Influenza A virus is found to selectively induce ATF6 translocation to promote caspase‐12‐dependent cell apoptosis.^67^ The respiratory syncytial virus (RSV) activates a non‐canonical ER stress response with preferential activation of the IRE1 and activated ATF6 pathways without concomitant significant activation of the PERK pathway.^68^

**Table 1:** Viruses capable of modulating the unfolded protein response

Name	Mechanism	References
Influenza A virus	Induces ER stress for upregulation of ER‐resident protein ERp57 to facilitate viral protein cleavage	84
Human cytomegalovirus	Encodes a protein, pUL38, which phosphorylates PERK and suppresses IRE1 to prevent cellular apoptosis induced by ER stress, limiting immune detection	58, 66
Hepatitis B virus	Activates ATF6 and IRE1/sXBP1 pathways of the UPR	59, 85
Hepatitis C virus	Activates IRE1 but inhibits XBP1 and blocks eIF2α phosphorylation to prevent upregulation of ERAD machinery in order to establish persistency in infected hepatocytes	57, 86, 87
West Nile virus	Induces CHOP (UPR transcription factor) to induce apoptosis	88
Bovine viral diarrhoea virus	Induces CHOP to induce apoptosis	63
Respiratory syncytial virus	Induces caspase‐12‐dependent apoptosis through activating the UPR; Preferential activation of the IRE1 and activated ATF6 pathways with no concomitant significant activation of the PERK pathway	68, 89
Dengue virus	Activates the UPR to facilitate protein folding but not to induce apoptosis	90
Japanese encephalitis virus	Induces CHOP to induce apoptosis in fibroblasts and neuronal cells	91
Chikungunya viruses (CHIKV)	CHIKV non‐structural protein 4 (nsp4) expression in mammalian cells suppresses eIF2α phosphorylation that regulates the PERK pathway	92
Severe acute respiratory syndrome coronavirus (SARS‐CoV)	The 8ab protein binds directly to the luminal domain of ATF6, the type II ER stress sensor, to induce UPR activation.	93
African swine fever virus	Maintain eIF2α phosphorylation independent of PERK activation; ASFV is capable of blocking the expression of CHOP	94

Autophagy is believed to be an ancient anti‐viral response. Upon evading the host, viral proteins can often be targeted by host autophagy pathway for lysosomal degradation, which promotes anti‐viral innate and adaptive immunities via facilitating viral protein processing and presentation to antigen‐presenting cells.^69^ It is recognised now that the autophagy pathway can play both anti‐viral and pro‐viral roles in the pathogenesis of different viruses. Some viruses can utilise autophagy proteins to foster their own growth intracellularly. Under such conditions, autophagy pathways are often served as scaffold for viral entry or served as inter‐cellular organelle to foster viral replication, suppressing innate immunity through insufficient viral protein presentation and preventing cell death.^70^ The immune system has developed alternative ways to interrupt viral protein synthesis. These include upregulation of inflammatory cytokines, such as interferons, to reinforce cellular stress and autophagy response to combat viruses.

### Evolution of cytokine‐driven interruption in protein biosynthesis

**Figure 5:**
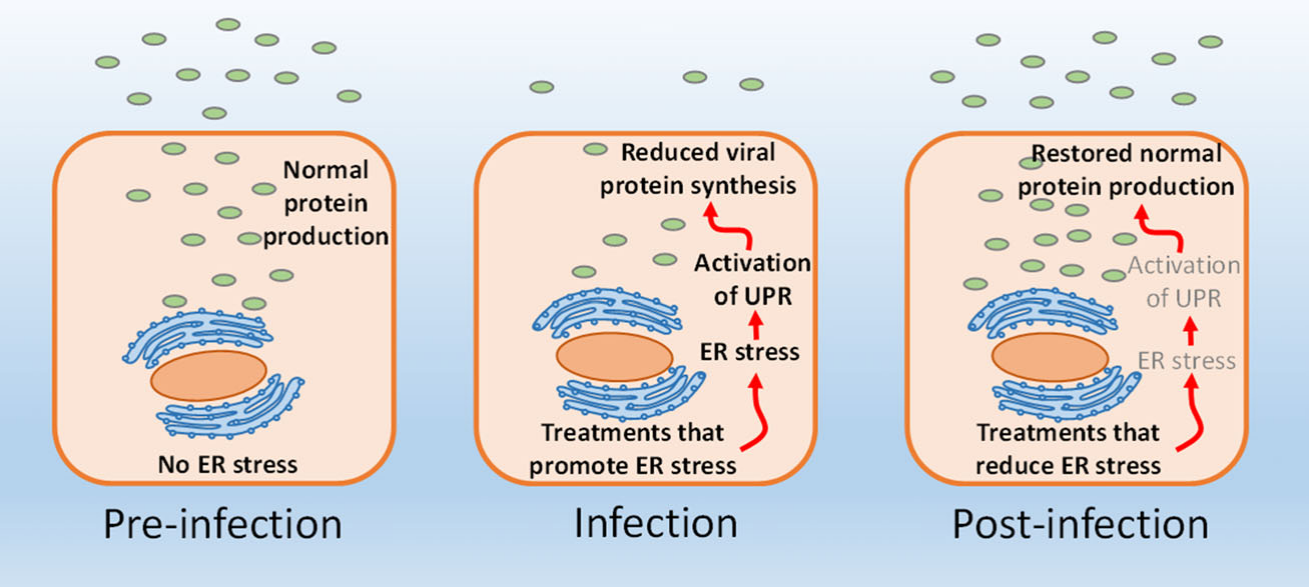
Proposed mechanism of anti‐viral function of cytokines. *Pre‐infection*: Basal level of UPR maintains the homeostasis of secretory cell function. *Infection:* Host ER stress and UPR are intrinsic mechanisms that will limit viral protein synthesis. However, viruses can potentially bypass these mechanisms to replicate. Specific cytokines (such as type I IFNs) that induce oxidative stress would lead to protein misfolding which will be beneficial in limiting viral infection. *Post‐infection:* Following viral clearance, the wound healing pathways are activated. Specific cytokines, such as IL‐10, known to suppress UPR signals and ER stress, will allow the restoration of normal protein production in the cells.

Our discovery of cytokine‐induced oxidative stress (ROS/NOS) within target cells provides a mechanism that bypasses this viral‐driven mechanism that block arms of the UPR. Innate cytokines are released quickly and dynamically. We identified selected cytokines that have a direct downstream chemical effect within the ER, which induces misfolding of viral proteins regardless of any viral blocking of the UPR (Figure 5). Translated viral proteins will misfold due to the inappropriate oxidative state in the ER, leading to host cell apoptosis and reduced virulence.

The impact of cytokines on oxidative stress and the UPR can dictate protein biosynthesis. For example, type I interferon signalling has been shown to regulate chronic hepatitis B virus infection via induction of oxidative stress in epithelial cells.^71^, ^72^, ^73^, ^74^ Anti‐viral effects of other cytokines such as TNF‐α have also been shown to induce apoptotic pathways in infected cells. Our work shows that specific cytokines (IL‐17, IL‐23) locally cause ER stress, particularly in cells with an inherent susceptibility to protein misfolding or inadequate UPR.

As discussed, cytokines such as IL‐4, IL‐13 and IL‐10 can boost protein production in mucin‐secreting goblet cells.^54^, ^75^, ^76^ There are several reports of correlations of cytokine levels with disease; however, limited research has been done on the direct effect of cytokines on protein biosynthesis.

### Inappropriate activation of cytokine‐induced ER stress in chronic inflammation

While cytokines that amplify stress may be favorable in the context of halting viral replication, inappropriate or continued activation of this pathway in the absence of overt pathogens in chronic inflammatory diseases perpetuates a cycle of stress and inflammation.^77^ This could result in poor barrier function, due to reduced secretion of barrier proteins and diminished epithelial integrity (Figure 5). For example, type I IFNs are essential for clearing viral infection. However, prolonged activation of type I IFNs in chronic infection leads to immune dysfunction and exacerbated tissue damage in the respiratory tract.^77^, ^78^ A careful balance is required; inducing ER stress and UPR pathways will limit viral replication and promote viral clearance in acute infection. However, prolonged ER stress and UPR activation is detrimental to mucosal barrier regeneration and renewed homeostasis.

IL‐10 is an example of the multifaceted role a single cytokine can have on viral infection and the UPR. IL‐10 suppresses B‐cell antibody responses and T‐cell immunity; thus, its neutralisation leads to viral clearance.^79^ Human cytomegalovirus has been found to encode a decoy IL‐10‐like cytokine, which signals through the human IL‐10 receptor to circumvent detection and destruction by the host immune system.^80^ We have demonstrated that IL‐10 suppresses the UPR in secretory goblet cells in the intestine,^54^ allowing for increased protein production. Therefore, along with IL‐10‐mediated immunosuppressive effects on the immune cells, IL‐10‐induced protein production (via the regulation of the UPR) would allow for increased viral replication in these highly productive secretory cells. Despite the negative effects of IL‐10 in acute viral infection, the importance of IL‐10 in attenuating chronic inflammation is unequivocal.^81^, ^82^ Regardless, these cytokines controlling secretory protein synthesis in non‐immune cells are an overlooked paradigm in immunity. More detailed understanding will help identify novel targets that can be modulated in acute infection and more broadly in chronic inflammatory diseases to alleviate tissue damage.

## Implications for therapy against viral replication and chronic pathology

Endemic and emerging viral infections cause profound morbidity and mortality, with over 13 500 hospitalisations and more than 3000 deaths per year in Australia due to influenza alone.^83^ Pathology in susceptible individuals is likely to involve either an inability to control the viral replication and dissemination, or the development of a local ‘cytokine storm’ damaging the mucosal epithelium. Better understanding of the contribution of ER stress‐associated UPR response in viral infection process, temporal sequence of events and pathways to initiation of chronic inflammation and tissue damage is imperative. Understanding how immune responses and inflammatory cytokines regulate UPR in viral infection is crucial, because boosting this physiological process via cytokines could open the way to novel approaches to limit viral replication and acute disease, and rescue individuals with persistent inflammation and associated tissue damage. Clearly, the UPR is intimately involved in viral replication: manipulating ER stress and UPR signalling could potentially limit viral replication and attenuate acute viral infections. Reduction in epithelial cell UPR activation in chronic disease provides an opportunity to limit persistent mucosal inflammation and the extent of tissue damage. The timing and nature of the cytokine response dictate time taken for both viral clearance and restoration of mucosal integrity and homeostasis. Manipulation of cytokine‐induced epithelial cell ER stress gives us a chance to tip this fine balance in favor of health rather than disease.

## Conflict of interest

The authors declare that there is no conflict of interest associated with this manuscript.
